# The Degradation Process of Typical Neonicotinoid Insecticides in Tidal Streams in Subtropical Cities: A Case Study of the Wuchong Stream, South China

**DOI:** 10.3390/toxics11030203

**Published:** 2023-02-22

**Authors:** Qunpo Jia, Yanpeng Cai, Xiao Yuan, Bowen Li, Bo Li

**Affiliations:** 1 Guangdong Provincial Key Laboratory of Water Quality Improvement and Ecological Restoration for Watersheds, Institute of Environmental and Ecological Engineering, Guangdong University of Technology, Guangzhou 510006, China; 2 Key Laboratory for City Cluster Environmental Safety and Green Development of the Ministry of Education, School of Ecology, Environment and Resources, Guangdong University of Technology, Guangzhou 510006, China

**Keywords:** neonicotinoid insecticides (NEOs), degradation kinetics, urban tidal stream, mathematical modeling, central composite design (CCD)

## Abstract

Neonicotinoid insecticides (NEOs) are commonly used to prevent unwanted insects in urban fields. Degradation processes have been one of the important environmental behaviors of NEOs in an aquatic environment. In this research, hydrolysis, biodegradation, and photolysis processes of four typical NEOs (i.e., thiacloprid (THA), clothianidin (CLO), acetamiprid (ACE), and imidacloprid (IMI)) were examined through the adoption of response surface methodology–central composite design (RSM-CCD) for an urban tidal stream in South China. The influences of multiple environmental parameters and concentration levels on the three degradation processes of these NEOs were then evaluated. The results indicated that the three degradation processes of the typical NEOs followed a pseudo-first-order reaction kinetics model. The primary degradation process of the NEOs were hydrolysis and photolysis processes in the urban stream. The hydrolysis degradation rate of THA was the highest (1.97 × 10^−5^ s^−1^), and that of CLO was the lowest (1.28 × 10^−5^ s^−1^). The temperature of water samples was the main environmental factor influencing the degradation processes of these NEOs in the urban tidal stream. Salinity and humic acids could inhibit the degradation processes of the NEOs. Under the influence of extreme climate events, the biodegradation processes of these typical NEOs could be suppressed, and other degradation processes could be further accelerated. In addition, extreme climate events could pose severe challenges to the migration and degradation process simulation of NEOs.

## 1. Introduction

Neonicotinoids (NEOs) are a class of widely used systemic insecticides, with usage registration in 120 countries for targeting over 140 crops [1]. Presently, NEOs are considerably used and occupy a quarter of the global insecticide market [1,2]. NEOs are used in public health and residential applications, such as pet flea treatment, horticulture, and household pest control products [3]. The overuse of NEOs has led to near-ubiquitous environmental detection in urban areas, particularly in urban streams all over the world [4,5,6,7]. Detection in surface water and sediment has been reported in the Guangzhou section of the Pearl River, with concentrations as high as 321 ng/L and 2.59 ng/g, respectively [8]. Traditional activated sludge treatment has exhibited an unsatisfactory removal of NEOs, resulting in treated wastewater being considered a potential source [9,10,11]. These phenomena also illustrate that NEOs have low abiotic degradation rates in aquatic environments. Degradation processes in an aquatic environment determine the environmental fates and ecological risks of NEOs. Previous studies have investigated the multiple degradation paths of different NEOs [12]. Data applied to the natural water environment have rarely been reported, especially in urban streams.

**Figure 1:**
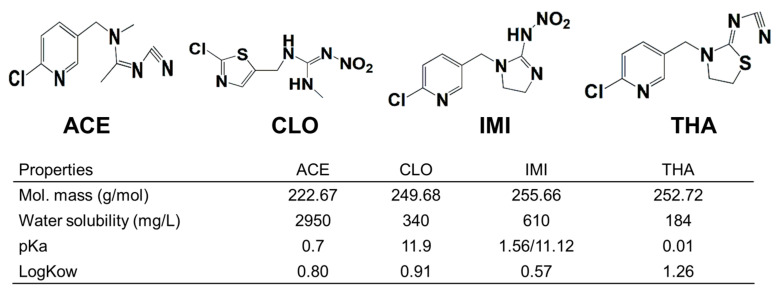
Molecular structures (top) and some chemical properties of ACE, CLO, IMI, and THA, respectively. Data are from PubChem (https://pubchtm.ncbi.nlm.nih.gov/, accsess on 5 October 2022) and PPDB (http://sittm.htrts.ac.uk/atru/ppdb/tn/indtx.htm, accessed on 1 September 2022).

Previous studies demonstrated that NEOs undergo two major degradation processes in surface water: hydrolysis and photolysis [13]. Biotic degradation also contributes to the environmental behavior and transformation of NEOs [14,15]. Hydrolysis processes of NEOs essentially involve a nucleophilic substitution reaction in which nucleophiles (i.e., H2O and OH^−^) attack the electrophilic groups (i.e., C, S, and P) in the NEO molecules [16]. The extent of hydrolysis depends on pH, temperature, dissolved organic carbon concentration, and humic acid content [13,17,18]. Photochemical degradation contributes to the environmental fate of NEOs in surface water and always occurs via direct or indirect photolysis [19]. Multiple environmental factors function as photosensitizers, such as humic acids, CO_3_^−^, OH^−^, and inorganic materials [19]. NEOs react via direct photolysis in both ultrapure and natural aqueous solutions, resulting in the half-lives of NEOs ranging from hours to days [20]. Bioremediation has been considered a cost-effective and eco-friendly approach for the treatment of pesticide-polluted environments. Previous literature reported that certain NEO-degrading microorganisms were isolated and characterized [21]. In addition, different NEOs would exhibit different degradation kinetics in the same degradation processes, which could be influenced by molecular structures and chemical properties (as indicated in Figure 1). The photodegradation rates of dinotefuran, imidacloprid, and thiamethoxam were 0.20, 0.30, and 0.18 mg∙L^−1^∙h^−1^, respectively [22,23,24]. Previous degradation experiments mostly simulated and optimized the degradation processes of NEOs in the laboratory by single-factor experiments, ignoring the multiple natural environmental factors.

Due to the humid/hot climate and their wide use, high levels of NEOs have been detected in urban streams in Guangzhou [8]. Extreme weather events such as heat waves, floods, or storms have increased in frequency over recent decades in Guangzhou, China. Studies have reported that extreme weather events could affect water quality and aquatic biota [25,26]. Other studies have also noted that extreme weather events affected the degradation processes of emerging contaminants [27,28]. Few data are available on climate change’s direct and indirect effects on the degradation processes of NEOs in urban streams.

In this study, water samples collected from an urban tidal stream in Guangzhou, China, were used as substrates. IMI, CLO, ACE, and THM were used as typical NEOs. The hydrolysis, biodegradation, and photolysis processes of these typical NEOs were assessed by considering multiple environmental factors and concentration levels. The degradation processes of influencing factors were examined by the RSM-CCD method. The main objectives of this study were (1) to research the degradation processes of typical NEOs and associated key influence factors in urban tidal streams, (2) to establish the kinetic equations reflecting the degradation of typical NEOs in the target aquatic environments, and (3) to predict the degradation processes and rates of typical NEOs under extreme climate conditions.

## 2. Materials and Methods

### 2.1. Chemicals and Reagents

Four standard NEOs and two internal standards (IMI-D4 and CLO-D3) were purchased from Dr. Ehrenstorfer GmbH (Augsburg, Germany) with a purity higher than 98%. The molecular structure and some chemical properties of these NEOs are shown in Figure 1. NEO stock solutions of the six standards were prepared by dissolving each compound in methanol (500 ppm) and were then maintained at −20 °C. HPLC-grade methanol and formic acid (98%) were provided by Aladdin (Shanghai, China). Deionized water was obtained from a Millipore purification system (CN120RDM1-230, VEOLIA, Shanghai, China). Other agents were all purchased from Aladdin (Los Angeles, CA, USA), such as hydrochloric acid (HCl), sodium hydroxide (NaOH), sodium chloride, humic acid (fulvic acid, 90%), and disodium ethylenediamine tetraacetate (Na2EDTA).

### 2.2. Water Collection and Pretreatment

Water samples were collected in 20 L polypropylene bottles at a 50 cm depth downstream of an urban tidal stream in Guangzhou, China. Some water indicators are shown in Table S1. Regarding the hydrolysis and photolysis processes, the raw waters were filtered through 0.45 and 0.22 μm cellulose nitrate filters to remove any particles and microorganisms. The filtered water samples were stored in the dark at 4 °C for 3 days, which inhibited microbial growth and restrained NEO biodegradation [29]. The unfiltered raw water samples were employed in the biodegradation analysis of NEOs. Five g/L Na2EDTA was used to reduce the interference of metal ions in the water samples.

### 2.3. Central Composite Design of Three Degradation Experiments

The response surface methodology (RSM) was an effective method for experimental process modeling and optimization [30]. Central composite design (CCD) was widely used in the RSM experiment. A five-level fractional design was also the most frequently used for the construction of second-order RSM models [31]. The CCD method was employed to optimize multiple parameters through systematic variation in all variables in a well-designed experiment with the minimum but required number of experiments. The degradation experiments were divided into three reaction processes (biodegradation, photolysis, and hydrolysis processes). Multiple environmental factors were carefully selected, including the initial concentration, temperature, pH, salinity, humic acid content, degradation time, and irradiance.

The hydrolysis and biodegradation experiments were conducted under the same experimental conditions as in the degradation experiments. The solution pH was adjusted with HCl or NaOH and monitored by a SevenEasy S20K pH meter fitted with a combined glass electrode. A rotary photochemical reactor (BL-GHX-V, Bilon, Shanghai, China) with a solar-simulated light source (a 1000 W xenon lamp with 290 nm filter, λ > 290 nm) was used for the photolysis experiments in a dark room (Figure S1). Circulating cooling water was used to control and maintain the temperature in the degradation experiments [32,33].

The ranges and levels of the selected environmental variables are listed in Table 1. The hydrolysis and biodegradation experiments were conducted under the same experimental conditions. According to the CCD design in the software Design Expert, hydrolysis and biodegradation simulation experiments needed 40 experimental samples, while the photolysis simulation experiment needed 50 experimental samples.

### 2.4. Sample Preparation and Extraction

Each 200 mL water sample was extracted using Oasis HLB SPE cartridges (200 mg, 30 μm, water). The internal standard (CLO-d3,500 ppb) was added to water samples as a surrogate standard to determine the recovery. After extraction, the SPE cartridges were rinsed with 10 mL deionized water, dried under vacuum for 20 min to remove water, and eluted with 10 mL methanol (HPLC-grade). The target eluents were concentrated with nitrogen and diluted to 200 μL with methanol (HPLC-grade). Furthermore, 50 μL of the internal standard (IMI-D4, 500 ppb) was added, and the final extracts were mixed. The internal standard was used as an internal reference for qualitative analysis [34]. Finally, the final eluents were filtered with 0.22 μm aqueous-phase filter membranes into internal intubations in liquid-phase automatic injection vials.

### 2.5. Instrumental Analysis and Quality Control

The target eluents were determined using an LC-MS/MS system (QTEAP5500, AB SCIEX, Oregon, USA) with the electrospray ionization positive mode. The gradient elution program, multiple reaction monitoring transition (MRM), collision energy (CE), and retention time (RT) of the analyzed NEOs are summarized in Table S2. A total of 5 μL of the final concentrations was injected into the chromatographic column (XBridge BEH C18 XP column (2.5 µm, 3 × 100 mm, 1/PKG)). The column temperature was maintained at 40 °C. The mobile phase was 0.1% formic acid water (a) and acetonitrile (b). The flow rate was 300 μL/min, as indicated in Table S3. 

The determination of NEOs was quantified with internal standard calibration [35]. The analysis method in terms of linearity was verified according to the correlation between the internal standard and the standard concentrations and peak areas of the analytes. The results indicated that the satisfactory correlation coefficients (R) of this method were higher than 0.99. The limit of detection (LOD) and the limit of quantification (LOQ) were determined as 3 and 10 times the signal-to-noise ratio (S/N), respectively. The LOQs of the four target NEOs varied between 0.09 and 0.15 ppb. The recovery of the method ranged from 92.5 to 102.3% (as shown in Table S4).

### 2.6. Data Analysis

Experimental design and mathematical modeling were performed in Design-Expert 8.0. A significant difference was determined as *p* < 0.05. One-way ANOVA was used in the analysis of the experiment data. The Nlinfit function was used for numerical fitting in MATLAB 2017. 

In this study, the degradation parameters of the typical NEOs were determined using the first-order reaction kinetic model in Equation (1) [36], as follows:

(1) $$C_{t}=C_{0}\times e^{-kt}$$

where C_0_ and *C_t_* are the concentrations of the NEOs at times 0 and *t*, respectively; *k* is the degradation rate constant (s^−1^); and t is the experiment time (s).

The half-life (*t*_1/2_) was computed with Equation (2) as:

(2) $$t_{1/2}=\frac{\ln\left(2\right)}{K}$$

where K is the degradation rate constant (s^−1^)

The degradation processes of NEOs were affected by various environmental factors. In this study, the multiple linear regression equation was used for the response surface regression procedure of CCD, as follows in Equation (3):

(3) $$C_{NEOs}=\alpha +\overset{k}{\underset{i=1}{\sum }}\beta_{i\;}X_{i}+\overset{k}{\underset{i=1}{\sum }}\overset{k}{\underset{j=1}{\sum }}\beta_{ij}X_{i}\;X_{j}+\overset{k}{\underset{i=1}{\sum }}\gamma_{i\;}X_{i}^{2}$$

where $C_{NEOs}$ is the concentration of each typical NEO at time t in every degradation process; $X_{i}$ and $X_{j}$ are the selected variables; α is a constant coefficient; $\beta_{i}$ is a coefficient determining the influence of each parameter $X_{i}$ in the response; $\beta_{ij}$ is a coefficient referring to the effects of the interactions among independent variables; and $\gamma_{i}$ is a coefficient capturing the quadratic effects of each parameter $X_{i}$.

## 3. Results and Discussion

### 3.1. Hydrolysis

**Figure 2:**
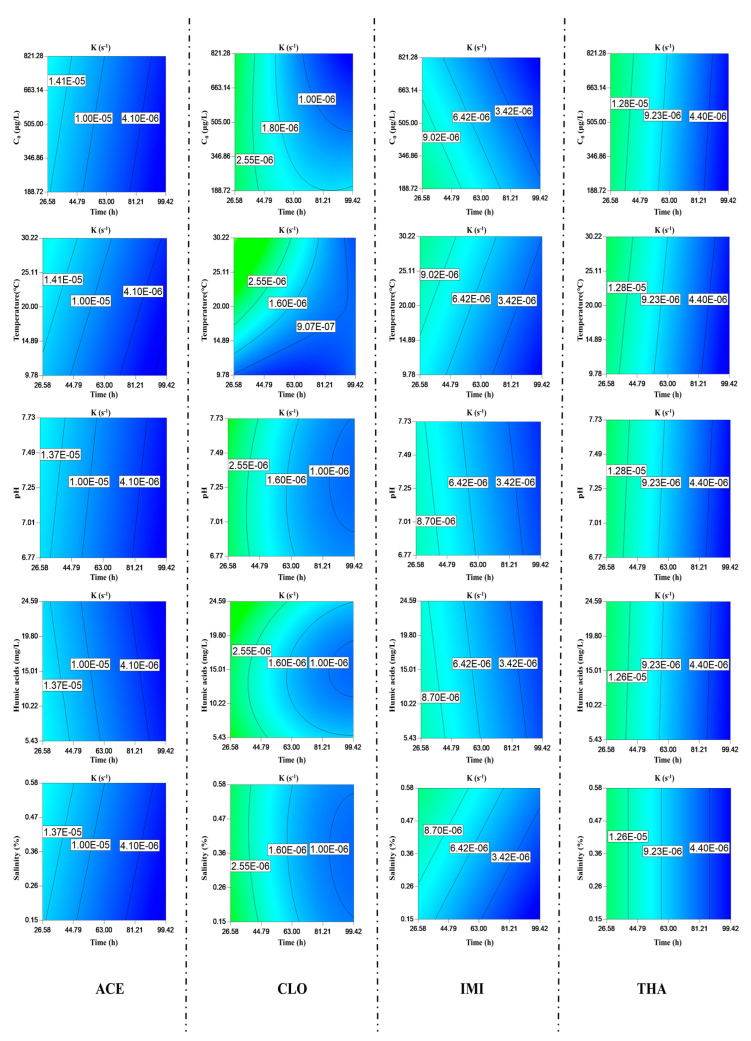
Hydrolysis of four typical NEOs and environment parameters affecting NEO hydrolysis.

The hydrolysis of the typical NEOs is presented in Figure 2. The hydrolysis rates of NEOs were influenced by the initial concentrations. Over time, the hydrolysis rate of ACE was higher than that of other NEOs. The amount of hydrolyzed ACE was close to 20% of the initial concentration. The hydrolysis rate of CLO was negatively correlated with the initial concentration. As the initial concentrations increased, the hydrolysis rate of CLO decreased. When the concentrations of CLO grew from 188.72 to 821.28 ng/L, the hydrolysis rate decreased from 2.0 × 10^−6^ s^−1^ to 1.23 × 10^−6^ s^−1^. In addition, temperature and pH can significantly influence NEOs’ hydrolysis processes. It was evident that as the temperature increased from 9.78 to 32.22 °C, the hydrolysis rates of CLO increased from 3.22 × 10^−7^ s^−1^ to 2.41 × 10^−6^ s^−1^. A similar situation was observed from other NEOs. The hydrolysis rate of ACE was increased by 13% when the temperature was increased by 10 °C. The hydrolysis rate of THA was increased by 2.6% under the same condition. The hydrolysis of NEOs was an endothermic reaction, as an increase in temperature could accelerate the hydrolysis process. NEOs were preferentially hydrolyzed under alkaline conditions than acid conditions. For example, the hydrolysis rates of THA increased from 8.18 × 10^−6^ to 8.83 × 10^−6^ s^−1^, with pH rising from 6.77 to 7.72. These findings are consistent with Todey [36], who reported the accelerated hydrolysis of some NEOs with increasing pH.

Other environmental parameters also affected the hydrolysis processes of some NEOs. Salinity seemed to promote the hydrolysis of ACE and IMI. With the increase in salinity, IMI and ACE hydrolysis rates increased by 10% and 5%, respectively. There were no apparent effects of the salinity on the hydrolysis rates of CLO and THA. Humic acids imposed a slight enhancement effect on the hydrolysis of THA but inhibited that of IMI and ACE. The hydrolysis rate of CLO increased from 8.28 × 10^−6^ s^−1^ to 8.74 × 10^−6^ s^−1^ when the humic acid concentration was increased from 5.43 to 24.59 mg/L. 

Forty designed runs of experimental conditions were derived from CCD. These experimental conditions and their results were summarized in Table S5. The analysis of variance (ANOVA) was listed in Table S6. The model F value indicated that the model was very significant (28.53, 15.46, 22.14, and 6.82). In this study, the significance of each coefficient was assessed based on p-values, and the *p*-values of the model were all <0.0001. Low *p*-values always indicate a higher significance of the corresponding parameter in the regression model. The quality of the model was statistically evaluated based on the test values. The obtained coefficients of determination suggest that the models fit the experimental data well (R^2^ = 0.96, 0.95, 0.94, and 0.854, respectively). In other studies, the Adep precision value was considered, which reflects the signal-to-noise ratio and a value greater than 4 [37]. In this study, the Adep precisions of four NEOs indicate that these models exhibited a sufficient resolution to reflect the experimental space. The response variable values matched the reduced cubic model equation well. The hydrolysis rates of NEOs were as expressed in Equations (S1)–(S4).

Regarding the organic compounds, there existed a quantitative relationship between the hydrolysis rate constant and temperature, and the hydrolysis rate could increase with increasing temperature. Furthermore, rate constants vary as a function of the temperature according to the Arrhenius equation [38]. 

An Arrhenius correction parameter (Equation (4)) was used in this research:

(4) $$K_{w}=A\times T^{m}e^{-\frac{E_{a}}{RT}}$$

where *K_w_* is hydrolysis rates of NEOs (s^−1^); T is the absolute temperature (K); T is the absolute temperature (K); A is referred to as the former factor, also known as the Arrhenius constants, with the same units as these of K; *m* is the coefficient for temperature correction; $E_{a}$ is the activation energy ($\mathrm{KJ}\cdot {\mathrm{mol}}^{-1}$); and *R* is the universal molar gas constant, i.e., 8.314 ($J\cdot {\mathrm{mol}}^{-1}\cdot K^{-1})$.

As indicated in Equations (1) and (4), $K_{w}$ was the pseudo-first-order hydrolysis rate constant of NEOs. After applying the environmental factors, the Kw values of the target NEOs were 1.97 × 10^−5^ s^−1^ (THA), 1.28 × 10^−5^ s^−1^ (CLO), 1.33 × 10^−5^ s^−1^ (ACE), and 1.53 × 10^−5^ s^−1^ (IMI). The temperature correction coefficients were −1.91 (CLO), −2.8 (ACE), −2.49 (IMI), and −3.03 (THA).

### 3.2. Photolysis

**Figure 3:**
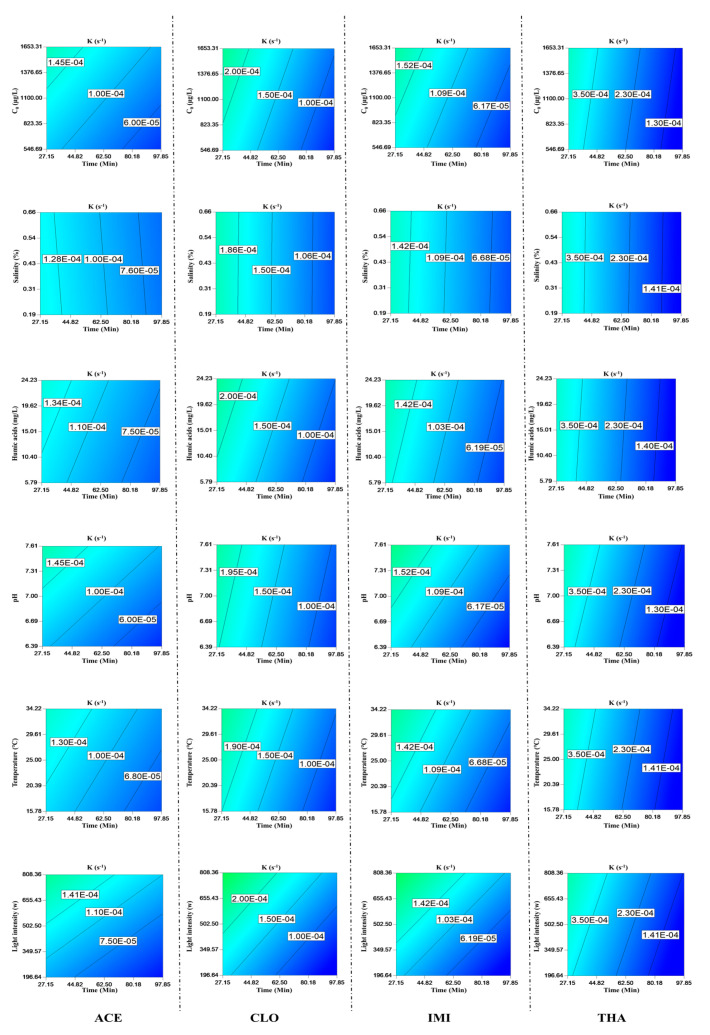
Photolysis of four typical NEOs and environment parameters affecting NEO photolysis.

As shown in Figure 3, the photolysis rates of NEOs increased with increasing temperature. The maximum photolysis rate of the NEOs was observed for IMI at 12% from 15 to 35 °C. In theory, the temperature might affect both direct and indirect photolysis processes. With increasing temperature, the quantum yields of organic contaminants, including NEOs, could also increase. Moreover, the type and rate of active species (ROS) could increase with the temperature, such as ·OH^−^ and H_2_O_2_. Due to temperature changes in urban streams, NEOs also had different half-lives in surface water from upstream to downstream.

NEOs were sensitive to photolysis and more easily degraded under alkaline conditions when the solution pH was increased. The pH was the dominant parameter for ACE, with a more significant impact than that observed for the other NEOs: the same increase in pH produced a decrease of 10% in concentration. We also found that photolysis rates of IMI increased from 7.23 × 10^−5^ s^−1^ to 1.35 × 10^−4^ s^−1^ when pH increased from 6.39 to 7.61. Humic acids could promote NEOs’ photolysis. Salinity could change NEOs’ photolysis process, but the effects were nonsignificant. When salinity increased from 0.19 to 0.66%, the photolysis rates of IMI increased from 1.02 × 10^−4^ s^−1^ to 1.05 × 10^−4^ s^−1^. When salinity rose from 0.19 to 0.66%, the photolysis rates of CLO increased from 1.04 × 10^−4^ s^−1^ to 0.99 × 10^−5^ s^−1^. 

The photolysis of NEOs also followed the pseudo-first-order degradation model, as expressed in Equation (1), and many environmental factors could impact NEO photolysis. To simplify the photolysis rate to the selected environmental factors, the response variable results matched the modified model best. The photolysis rates of NEOs were as expressed in Equations (S5)–(S8). Fifty designed runs of experimental conditions were derived from the CCD method. These experimental conditions and their results were summarized in Table S7. The analysis of variance (ANOVA) was listed in Table S8. The model F values of the photolysis equations were 12.17, 13.54, 12.25, and 17.52. The *p*-values were all below 0.01. These values all indicated that the overall models were significant. Among the selected environmental factors, temperature, photolysis time, and light energy contributed the most to NEO photolysis processes (*p* < 0.001). The pH value was also an important parameter because it could affect the photolysis of certain NEOs, such as ACE and THA (*p* < 0.0001), IMI (*p* < 0.0003), and except CLO (*p* > 0.05). No significant NEO photolysis effects of salinity and humic acids were observed in this study (*p* > 0.05). Thus, the corresponding experimental data were used in the above equations. 

In a virtual environment, pH, temperature, and light intensity were essential parameters influencing the photolysis process of NEOs in surface water. Thus, Equation (1) was refined to fit the actual environment [31], as expressed in Equation (5):

(5) $$K_{p}=2.303\times \alpha \times M\times e^{\;\gamma \cdot {\left(pH-pKa\right)}^{2}\times \left(T+273.15\right)}\times W$$

where $K_{p}$ is the photolysis rates of NEOs (min^−1^); M and pKa are the molecular molar masses (g/mol) and dissociation constants of the NEOs, respectively; α is the correction coefficient; γ is the correction factor of temperature; pH, T, and W were the pH values, temperature (°C), and solar radiation (W), respectively. 

The Nlinfit function was used to obtain Equation (5), and the results are listed in Table 2. The MSE values indicated that the simulation results suitably agreed with the actual situations. Moreover, the photolysis rate constants ($K_{p}$) of the target NEOs are also listed in Table 2. Based on the results, the half-lives of the typical NEOs differed, ranging from 14 to 200 min. These results indicate that the photolysis of CLO in surface water could occur much slower than other NEOs.

### 3.3. Biodegradation

**Figure 4:**
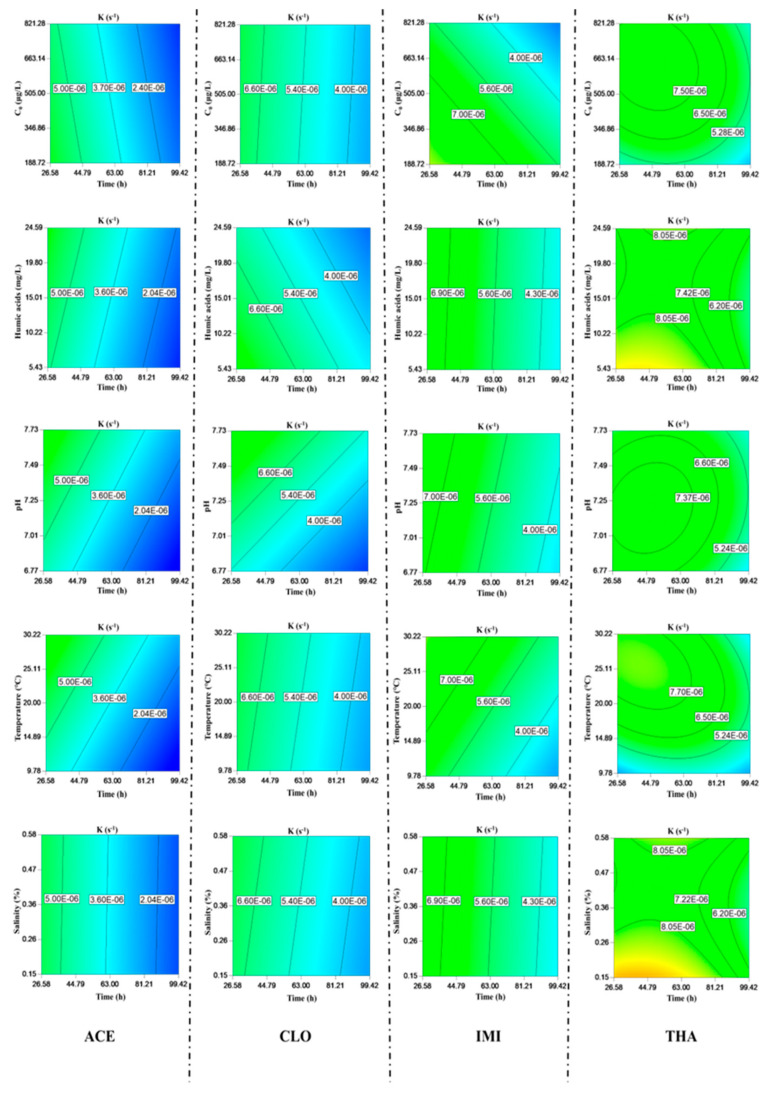
Biodegradation rates of four typical NEOs and environmental parameters affecting NEO biodegradation.

As shown in Figure 4, temperature and pH could influence the NEO biodegradation rate. Microbial degradative activities in surface water depend on the optimal growth pH and temperature. The effect of temperature on the biodegradation rate might be attributable to microbial growth and enzymatic activity. Bacterial growth was repressed under both acidic and alkaline conditions. ACE biodegradation increased from 35 to 52%, and that of CLO rose from 52 to 70% when pH increased from 6.77 to 7.73. The pH did not significantly affect the biodegradation processes of IMI and THA.

The biodegradation processes of THA and IMI were also affected by the initial concentration because these NEOs might be supplemented as carbon sources in the biodegradation process. Regarding CLO, the difference was even more negligible, with a slight influence on the biodegradation results since the initial concentration did not significantly influence the biodegradation rate. In addition, the biodegradation rates of ACE were the lowest among the four typical NEOs. This phenomenon indicates that its biodegradation was the slowest, especially at higher concentrations. 

Due to the restriction of microbial growth, NEO biodegradation in seawater was more difficult than in fresh water [14]. In this study, salinity slightly promoted the biodegradation of ACE and CLO but imposed a slight inhibitory effect on IMI and THA. Humic acids could enhance the biodegradation rates of ACE, IMI, and THA but inhibited that of CLO. The concentration of humic acids exerted different influences on the ACE biodegradation rate, and low concentrations of humic acids reduced the biological sensitivity. Humic acids represent a double-edged sword regarding NEO biodegradation. 

Forty designed runs of experimental conditions were also derived via the CCD method. The experimental conditions and results were provided in Table S9. The analysis of variance (ANOVA) results was listed in Table S10. The model F-value indicates that the model was very significant (6.0, 8.72, 6.92, and 9.70). The p-values of the model were all <0.0001. The quality of the models was statistically evaluated based on the test values, and the obtained coefficients of determination (R^2^ = 0.79, 0.88, 0.56, and 0.88) suggest that these models fit with the experimental data well. In the biodegradation processes of the typical NEOs, the Adep precision values (10.338, 10.297, 11.384, and 10.142) indicate that these models provided a sufficient resolution to reflect the experimental space properly. The response variable values fit the reduced cubic model equation of the biodegradation experiments well. The biodegradation rates of NEOs were as expressed in Equations (S9)–(S12).

The biodegradation processes of NEOs conformed to the first-order degradation kinetics, and the most suitable temperature for NEO biodegradation ranged from 22 to 37 °C [21]. The optimum biodegradation rate should be used in this experiment and can be calculated using Equation (6). Equation (6) was drawn from other literature [31,32].

(6) $$K_{B}=K_{best}\times e^{-\beta \times {\left(T-30\right)}^{2}}$$

where $K_{B}$ is the degradation rates of NEOs (s^−1^); $K_{best}$ is the pseudo-first-order biodegradation rate constants when the temperature was 30 °C; *T* is the water temperature (°C); and β is the shaping parameter. The $K_{best}$ values of ACE, CLO, IMI, and THA were 1.08 × 10^−7^, 5.75 × 10^−7^, 4.11 × 10^−7^, and 4.55 × 10^−8^ s^−1^, respectively. 

The Nlinfit function was employed to fit Equation (6), and the values of R were 3.12 × 10^−8^ (ACE), 1.67 × 10^−10^ (CLO), 2.88 × 10^−9^ (IMI), and 1.12 × 10^−7^ (THA). The shaping parameters (β) of the NEOs were 0.01 (ACE), 0.001 (CLO), 0.0046 (IMI), and 0.011 (THA).

The biodegradation rates of ACE, CLO, IMI, and THA were 4.86 × 10^−7^, 1.56 × 10^−6^, 1.11 × 10^−6^, and 1.23 × 10^−7^ (s^−1^), respectively. Moreover, the half-lives of the biodegradation processes in this study were determined by Equation (2). The half-lives were 16.51 days (ACE), 5.14 days (CLO), 7.23 days (IMI), and 65.22 days (THA). Due to different microbes and factors, the biodegradation rate differed among other streams [39]. The biodegradation percentage of CLO was only 37% in 37 days at 30 °C; the half-life of THA was 20.9 h; and 64.4% of IMI was degraded in 6 days in different water environments [40]. In addition, our result also proved why wastewater treatment systems could function as sources and sinks of NEOs in urban areas. Because the current municipal biological wastewater treatment process (such as the CASS and A2/O systems) attains an unsatisfactory removal of NEOs, almost 90% of untreated NEOs was discharged into urban rivers [41].

### 3.4. The Total Degradation of Typical NEOs in the Wuchong Stream

In this study, we assumed that there was no interaction between the hydrolysis, biodegradation, and photolysis processes of each NEO in the Wuchong Stream. The total degradation of each NEO also conformed to first-order reaction kinetics. Due to the riverbed of the Wuchong Stream being paved with rocks and sand, we neglected the adsorption–desorption processes at the multimedia interface, including water–air exchange. So, the total degradation of each NEO was as Equation (7):

(7) $$C_{t}=C_{0}\times e^{-kt}=C_{0}\times e^{-\left(k_{w}+k_{P\;}+k_{B}\right)t}$$

where $C_{0}$ is the initial concentration of NEOs; $C_{t}$ is the NEO concentrations at time *t* (s); *k* is the total concentration of each NEOs (/s); and the other variables are the same as above.

In this study, we analyzed the relationship between the NEOs degradation process and various environmental factors, such as temperature, pH, salinity, humic acid content, and solar radiation intensity. We discovered that temperature was the most critical factor impacting the three degradation processes. We also obtained the quantitative relationship between the temperature and the NEO hydrolysis, biodegradation, and photolysis processes. The three degradation processes and total degradation rates of NEOs were simulated under a certain temperature gradient by Equations (4)–(6). Based on Equation (7), the total degradation rate constant k of ACE was 2.14 × 10^−4^ s^−1^ in the Wuchong Stream, that of CLO was 1.66 × 10^−4^ s^−1^, that of IMI was 8.06 × 10^−4^ s^−1^, and that of THA was 7.32 × 10^−5^ s^−1^. In addition, among the four typical NEOs, THA exhibited the most prolonged half-life at 2.63 h, while CLO attained the shortest half-life at 0.24 h. 

**Figure 5:**
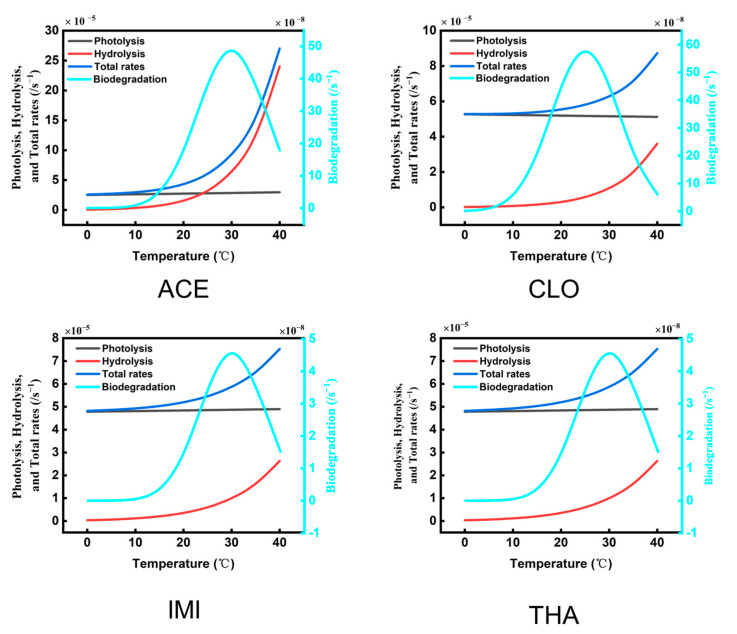
Effects of a single environment factor (temperature) on the degradation rates (total, photolysis, hydrolysis, and biodegradation rates) of the typical NEOs in the simulations.

As shown in Figure 5, the biodegradation rates of the typical NEOs were two orders of magnitude lower than the photolysis and hydrolysis. Therefore, the biodegradation process contribution accounted for only a very low proportion of the migration and transformation processes in the Wuchong Stream. In addition, due to the influence of solar radiation in the virtual natural environment, the photolysis process was the primary degradation process of the typical NEOs during the day, and hydrolysis was the primary process at night. Multiple environmental factors restrict and influence each other. 

The annual average temperature in the Greater Bay Area significantly increased at a heating rate of 0.18 °C∙(10a)^−1^ from 1961 to 2018. Studies reported that the annual sunshine hours significantly decreased at 60.1 h/10 a, and the yearly precipitation in Guangzhou increased over the past 58 years [42]. When the temperature increased from 20 to 30 °C, the hydrolysis rate constant of ACE also rose from 1.57 × 10^−5^ to 6.44 × 10^−5^ s^−1^, and the hydrolysis half-life of ACE decreased from 12 to 2.9 h. Under the same conditions, the hydrolysis half-life of IMI was also reduced from 1.3 to 0.4 h. The extremely high temperature increased from 31 to 38 °C, and the duration ranged from 191 to 241 days. The degradation rates of the NEOs increased by 210% (ACE), 124% (CLO), 196% (IMI), and 118% (THA). This high-temperature medium is not conducive to biodegradation and inhibits the activities of the microbial population. 

As climate change progresses, extreme weather events such as heatwaves and extreme rainfall could trigger factors as potential drivers of dengue outbreaks in Guangzhou [43]. This could lead to a surge in the residual concentrations of NEOs in urban streams. In addition, continuous high-temperature, and high-salinity environments of urban streams due to extreme climate events would pose severe challenges to the migration and degradation processes of NEO simulations.

## 4. Conclusions

This study explored the degradation processes of four typical NEOs (ACE, CLO, IMI, and THA) in an urban tidal stream using laboratory experiments with actual water samples. The three major routes of these typical NEOs were investigated with the CCD method, including hydrolysis, photolysis, and biodegradation. The total degradation rates of ACE, CLO, IMI, and THA were estimated at 2.14 × 10^−4^, 1.53 × 10^−4^, 8.06 × 10^−4^, and 7.32 × 10^−5^ s^−1^, respectively. In addition, THA attained the most extended half-life of 2.63 h, while the shortest half-life reached approximately 0.24 h. Photolysis and hydrolysis were the main degradation pathways of these NEOs in this urban tidal stream. It was also found that direct photolysis was the main photo-transformation pathway. The biodegradation processes of these NEOs were relatively neglected. In addition, the temperature was the main environmental factor influencing the degradation processes. Solar radiation also played an essential role in the photolysis processes of these NEOs. The extremely high temperature ranged from 31 to 38 °C, and the duration ranged from 191 to 241 days. The degradation rates of NEOs increased by 210% (ACE), 124% (CLO), 196% (IMI), and 118% (THA). Extreme climate events could affect the residue concentrations, degradation processes, and rates of NEOs in Guangzhou.

The natural water environment is complex and changeable. The degradation processes of NEOs are impacted by multiple environmental factors, not just the above factors. Furthermore, sediment is a potential source of NEOs, which also affects NEO migration and occurrence. In the future, we will examine the adsorption and desorption process of NEOs in sediment and establish a numerical model to depict the transportation and transformation of typical NEOs in an urban stream system.

## Figures and Tables

**Table 1:** Experimental parameters.

Environmental Factors	Units	−α ^c^	−1	0	1	α	Degradation Process
Initial concentration (C0)	ppb	10	233	505	776	1000	^a^
		200	525	1150	1675	2000	^b^
Time	h	6	32	63	94	120	^a^
	min	5	30	52.5	100	120	^b^
Temperature	°C	4	12	20	29	36	^a^
		10	15	25	34	40	^b^
Solar radiation	W	5	184	550	820	1000	^b^
Humic acid content	mg/L	0.02	5.79	15.01	24.23	30	^a/b^
pH		6.5	6.8	7.25	7.6	8	^a/b^
Salinity ^d^	%	0.05	0.18	0.425	0.66	0.8	^a/b^

^a^: Hydrolysis and biodegradation experiments; ^b^: photolysis experiment; ^c^: α = 1.62658 (axial point for orthogonal CCD in the case of 8 independent variables), and the actual values were rounded; and ^d^: the salinity value was expressed by the concentration of sodium chloride.

**Table 2:** Nlinfit-derived functions and photolysis rates of the target NEOs.

NEOs	α	γ	MSE	*K_p_* (min^−1^)
THA	8.318 × 10^−9^	1.202 × 10^−5^	8.64 × 10^−6^	0.0032
CLO	1.322 × 10^−8^	−4.26 × 10^−5^	3.58 × 10^−6^	0.091
ACE	1.901 × 10^−9^	1.04 × 10^−4^	1.56 × 10^−6^	0.012
IMI	1.55 × 10^−9^	1.4 × 10^−4^	1.60 × 10^−6^	0.0474

## Data Availability

Data are available upon request.

## Supplementary Materials

The following supporting information can be downloaded at: https://www.mdpi.com/article/10.3390/toxics11030203/s1, Figure S1: Rotary photochemical reactor; Table S1: The quality of water sample in Wuchong Stream; Table S2: Optimized LC-MS/MS parameters and retention times of target NEOs analyzed; Table S3: The gradient elution program of LC-MS/MS; Table S4: Linear range of matrix matched calibration curves for NEOs studied; Table S5: The results of target NEOs in hydrolysis degradation experiment; Table S6: Analysis of the variable table of target NEOs in hydrolysis degradation experiment; Table S7: The results of target NEOs in the photolysis degradation experiment; Table S8: Analysis of variable table of target NEOs in photolysis degradation experiment; Table S9: The results of target NEOs in biodegradation experiment; Table S10: Analysis of variable table of target NEOs in biodegradation experiment; Equations (S1)–(S4):The hydrolysis rates of NEOs; Equations (S5)–(S8):The photolysis rates of NEOs; Equations (S9)–(S12):The biodegradation rates of NEOs.
