# Supplementary material for: The Degradation Process of Typical Neonicotinoid Insecticides in Tidal Streams in Subtropical Cities: A Case Study of the Wuchong Stream, South China
Source: Toxics. 2023 Feb 22;11(3):203. doi: 10.3390/toxics11030203 (PMC10057386; doi:10.3390/toxics11030203)
Supplement: Supplementary file 1 [file toxics-11-00203-s001.zip › toxics-2176656-supplementary.pdf]

Supplementary Material

Spatial Distribution and Ecological Risks of Neonicotinoid Insecticides in an Urban Tidal Stream of Guangzhou City, South China

This file includes:

Table S1 The quality of water sample in Wuchong Stream
Table S2 Optimized LC-MS/MS parameters and retention times of target NEOs analyzed.
Table S3 The gradient elution program of LC-MS/MS.
Table S4 Linear range of matrix matched calibration curves for NEOs studied.
Table S5 The results of target NEOs in hydrolysis degradation experiment.
Table S6 Analysis of the variable table of target NEOs in hydrolysis degradation experiment.
Table S7 The results of target NEOs in the photolysis degradation experiment
Table S8 Analysis of variable table of target NEOs in photolysis degradation experiment.
Table S9 The results of target NEOs in biodegradation experiment
Table S10 Analysis of variabce table of target NEOs in biodegradation experiment.

Figure S1 Rotary photochemical reactor.

Equations (S1)–(S4):The hydrolysis rates of NEOs；
Equations (S5)–(S8):The photolysis rates of NEOs；
Equations (S9)–(S12):The biodegradation rates of NEOs.

Table S1. The quality of water sample in Wuchong Stream

Water sapmle	Ph	TDS	COD	TP	NO_2^-	NH_4-N	T	DO	Saility
	7.55	410	120.39	0.071	0.003	1.01	31.8	4.2	0.44

Table S2. Optimized LC-MS/MS parameters and retention times of target NEOs analyzed

Compound	MRM transition (m/z)	Fragment voltage (V)	Collision energy (eV)	Retention time (min)	Ionization mode
IMI	175~256.2	60	20	10.69	[M-H]+
	209~256.2	60	20	10.69	[M-H]+
ACE	126~223.1	130	20	10.99	[M-H]+
	187~223.1	130	20	10.99	[M-H]+
THA	126~253	130	25	11.61	[M-H]+
	186~253	130	25	11.61	[M-H]+
CLO	169~250	80	20	10.46	[M-H]+
	132~250	80	20	10.46	[M-H]+
IMI-d4	214~260.7	130	25	12.46	[M-H]+
	180~260.7	130	25	12.46	[M-H]+
CLO-d3	199~217	130	15	12.07	[M-H]+
	126~217	130	25	12.07	[M-H]+

Table S3. The gradient elution program of LC-MS/MS

Time	Module	Event	Parameter
0.2	Pumps	PumpB. Con.c	40
5.0	Pumps	PumpB. Con.c	90
6.0	Pumps	PumpB. Con.c	90
6.10	Pumps	PumpB. Con.c	40
10	System Controller	Stop	

Table S4. Linear range of matrix matched calibration curves for NEOs studied

Analytes	Range of curve (ng/L)	Calibration curve	*r*	LOD ng/L	LOQ ng/L	Average recovery (%)	Matrix effect (%)
CLO	0.1 ~ 1000	y = 20.13x+0.44	0.99822	0.03	0.09	92.5	0.12
IMI	0.1 ~ 1000	y = 3.50x-0.064	0.99822	0.04	012	102.3	1.53
ACE	0.1 ~ 1000	y = 14.15 x+ 0.47	0.99804	0.03	0.09	93.6	5.61
THA	0.1 ~ 1000	y =25.54 x+ 2.16	0.99878	0.05	0.15	94.1	0.87

Table S5. The results of target NEOs in hydrolysis degradation experiment

Run	Factor 1	Factor 2	Factor 3	Factor 4	Factor 5	Factor 6	C/C0			
	C0 (ppb)	Time (h)	Temperature (°C)	Ph	Salinity (%)	Humic acids (mg/L)	CLO	THA	IMI	ACE
1	1000.00	63.00	20.00	7.25	0.36	15.01	0.82	0.155	0.144	0.694
2	188.72	99.42	30.22	7.73	0.58	24.59	0.456	0.119	0.120	0.130
3	505.00	63.00	20.00	7.25	0.36	30.00	0.416	0.415	0.333	0.400
4	505.00	6.00	20.00	7.25	0.36	15.01	0.917	0.470	0.240	0.709
5	505.00	63.00	36.00	7.25	0.36	15.01	0.595	0.338	0.214	0.340
6	505.00	63.00	20.00	7.25	0.36	15.01	0.715	0.415	0.333	0.459
7	505.00	63.00	20.00	7.25	0.03	15.01	0.713	0.412	0.333	0.503
8	188.72	26.58	9.78	6.77	0.58	5.43	0.856	0.433	0.080	0.117
9	505.00	63.00	20.00	7.25	0.36	15.01	0.715	0.415	0.333	0.459
10	188.72	26.58	30.22	7.73	0.58	5.43	0.670	0.146	0.066	0.101
11	821.28	99.42	30.22	6.77	0.15	24.59	0.661	0.113	0.102	0.234
12	821.28	99.42	30.22	6.77	0.58	5.43	0.629	0.097	0.077	0.259
13	188.72	26.58	30.22	7.73	0.15	24.59	0.479	0.413	0.663	0.991
14	821.28	26.58	9.78	7.73	0.15	24.59	0.731	0.211	0.180	0.494
15	821.28	26.58	30.22	7.73	0.58	24.59	0.522	0.186	0.090	0.478
16	188.72	99.42	9.78	7.73	0.58	5.43	0.384	0.213	0.535	0.661
17	188.72	99.42	9.78	6.77	0.58	24.59	0.404	0.360	0.862	0.855
18	505.00	120.00	20.00	7.25	0.36	15.01	0.514	0.415	0.333	0.261
19	821.28	99.42	30.22	7.73	0.15	5.43	0.674	0.081	0.053	0.210
20	821.28	99.42	9.78	7.73	0.58	24.59	0.609	0.163	0.107	0.299
21	505.00	63.00	20.00	6.50	0.36	15.01	0.614	0.444	0.473	0.748
22	505.00	63.00	20.00	7.25	0.36	15.01	0.715	0.415	0.333	0.459
23	505.00	63.00	20.00	7.25	0.70	15.01	0.583	0.415	0.333	0.519
24	505.00	63.00	20.00	8.00	0.36	15.01	0.654	0.390	0.293	0.401
25	188.72	26.58	30.22	6.77	0.58	24.59	0.505	0.126	0.400	0.048
26	505.00	63.00	20.00	7.25	0.36	15.01	0.715	0.415	0.333	0.459
27	188.72	99.42	9.78	7.73	0.15	24.59	0.346	0.041	0.497	0.040
28	821.28	26.58	9.78	7.73	0.58	5.43	0.749	0.211	0.667	0.323
29	505.00	63.00	20.00	7.25	0.36	0.02	0.514	0.415	0.333	0.519
30	505.00	63.00	20.00	7.25	0.36	15.01	0.715	0.415	0.313	0.459
31	188.72	99.42	30.22	6.77	0.15	5.43	0.293	0.314	0.506	0.539

32	188.72	26.58	9.78	6.77	0.15	24.59	0.876	0.175	0.846	0.500
33	188.72	26.58	9.78	7.73	0.15	5.43	0.830	0.208	0.650	0.661
34	821.28	99.42	30.22	6.77	0.15	5.43	0.692	0.147	0.204	0.396
35	821.28	99.42	9.78	6.77	0.15	5.43	0.759	0.187	0.387	0.822
36	821.28	26.58	9.78	6.77	0.58	24.59	0.881	0.250	0.606	0.798
37	821.28	26.58	30.22	6.77	0.15	5.43	0.516	0.202	0.248	0.527
38	505.00	63.00	4.00	7.25	0.36	15.01	0.974	0.517	0.748	0.975
39	10.00	63.00	20.00	7.25	0.36	15.01	0.536	0.269	0.500	0.305
40	505.00	63.00	20.00	7.25	0.36	15.01	0.715	0.415	0.333	0.459

Table S6. Analysis of variable table of target NEOs in hydrolysis degradation experiment

Source	CLO		ACE		IMI		THA	
	F-Value	P-value	F-Value	P-value	F-Value	P-value	F-Value	P-value
Model	28.53	< 0.0001	22.14	< 0.0001	6.82	< 0.0001	15.46	< 0.0001
A-C0	53.89	< 0.0001	77.58	< 0.0001	1.24	0.2778	15.82	0.0010
B-Time	102.48	< 0.0001	4.47	0.00460	4.18	0.0536	7.89	0.0121
C-temperature	108.44	< 0.0001	87.58	< 0.0001	19.52	0.0002	8.66	0.0091
D-pH	0.032	0.8606	8.67	0.0075	5.53	0.0285	7.14	0.0161
E-Salinity	0.041	0.8412	4.68	0.0417	4.36	0.0491	0.0012	0.9147
F-Humic acids	0.60	0.4476	3.38	0.0795	0.33	0.5703	0.58	0.4558
R^2	0.9607		0.9448		0.854		0.9524	
Adep Precision	31.329		18.129		10.763		11.860	

Table S7. The results of target NEOs in photolysis degradation experiment

Run	Factor 1	Factor 2	Factor 3	Factor 4	Factor 5	Factor 6	Factor 7	C/C0			
	C0 (ppb)	pH	Salinity (%)	Temperature (°C)	Time (time)	Light energy (W)	Humic acids(mg/L)	CLO	THA	IMI	ACE
1	546.69	7.61	0.66	34.22	27.15	196.64	24.23	0.549	0.412	0.66	0.6
	1100.00	7.00	0.43	25.00	62.50	502.50	15.01	0.787	0.590	0.84	0.9
3	1100.00	7.00	0.43	25.00	62.50	502.50	15.01	0.787	0.590	0.84	0.9
4	1100.00	8.00	0.43	25.00	62.50	502.50	15.01	0.636	0.477	0.69	0.7
5	200.00	7.00	0.43	25.00	62.50	502.50	15.01	0.782	0.587	0.58	0.9
6	1653.31	6.39	0.19	34.22	97.85	196.64	5.79	0.596	0.447	0.63	0.7
7	1100.00	7.00	0.43	25.00	120.00	502.50	15.01	0.695	0.521	0.75	0.8
8	1653.31	6.39	0.66	15.78	97.85	196.64	5.79	0.763	0.572	0.80	0.9
9	1653.31	6.39	0.19	15.78	97.85	196.64	24.23	0.702	0.527	0.74	0.8
10	546.69	7.61	0.66	15.78	97.85	196.64	24.23	0.626	0.469	0.74	0.7
11	1100.00	7.00	0.43	25.00	62.50	502.50	15.01	0.787	0.590	0.84	0.9
12	546.69	6.39	0.66	34.22	27.15	808.36	24.23	0.659	0.494	0.77	0.7
13	546.69	7.61	0.19	34.22	97.85	808.36	5.79	0.465	0.349	0.58	0.5
14	1653.31	7.61	0.66	15.78	27.15	808.36	24.23	0.528	0.396	0.57	0.6
15	1653.31	6.39	0.66	34.22	97.85	196.64	24.23	0.591	0.443	0.63	0.7
16	546.69	7.61	0.19	15.78	27.15	808.36	24.23	0.674	0.505	0.78	0.8
17	1100.00	7.00	0.05	25.00	62.50	502.50	15.01	0.787	0.590	0.84	0.9
18	2000.00	7.00	0.43	25.00	62.50	502.50	15.01	0.780	0.585	0.81	0.9
19	1100.00	7.00	0.43	25.00	62.50	502.50	30.00	0.598	0.579	0.83	0.9
20	546.69	6.39	0.66	15.78	27.15	196.64	24.23	0.878	0.658	0.99	1.0
21	546.69	6.39	0.66	34.22	97.85	196.64	5.79	0.449	0.337	0.56	0.5
22	1100.00	7.00	0.43	25.00	62.50	1000.00	15.01	0.687	0.515	0.74	0.8
23	1100.00	7.00	0.43	40.00	62.50	502.50	15.01	0.649	0.487	0.70	0.7
24	1100.00	7.00	0.43	25.00	62.50	5.00	15.01	0.827	0.620	0.88	0.9
25	1653.31	7.61	0.19	15.78	27.15	196.64	24.23	0.813	0.610	0.85	0.9
26	546.69	6.39	0.19	15.78	97.85	808.36	5.79	0.772	0.579	0.88	0.9
27	1100.00	7.00	0.43	25.00	62.50	502.50	15.01	0.787	0.590	0.84	0.9
28	1653.31	6.39	0.66	34.22	97.85	808.36	5.79	0.476	0.357	0.51	0.5
29	1100.00	7.00	0.43	25.00	62.50	502.50	15.01	0.787	0.590	0.84	0.9
30	1100.00	7.00	0.43	25.00	5.00	502.50	15.01	0.930	0.697	0.98	0.9
31	546.69	6.39	0.19	34.22	97.85	808.36	24.23	0.503	0.377	0.61	0.6

32	1100.00	7.00	0.43	10.00	62.50	502.50	15.01	0.842	0.631	0.90	0.9
33	546.69	7.61	0.19	15.78	97.85	196.64	5.79	0.626	0.469	0.74	0.7
34	1653.31	7.61	0.19	34.22	27.15	808.36	24.23	0.395	0.296	0.43	0.4
35	546.69	6.39	0.19	34.22	27.15	196.64	24.23	0.852	0.625	0.94	0.9
36	1653.31	6.39	0.66	34.22	27.15	196.64	5.79	0.832	0.624	0.87	0.9
37	1653.31	7.61	0.19	15.78	27.15	808.36	5.79	0.516	0.387	0.55	0.6
38	546.69	6.39	0.19	34.22	27.15	808.36	5.79	0.662	0.497	0.77	0.7
39	1653.31	7.61	0.66	34.22	97.85	808.36	24.23	0.347	0.261	0.38	0.4
40	546.69	6.39	0.66	15.78	27.15	808.36	5.79	0.772	0.579	0.88	0.9
41	546.69	7.61	0.66	15.78	97.85	808.36	5.79	0.370	0.277	0.48	0.4
42	1653.31	7.61	0.19	15.78	97.85	808.36	24.23	0.407	0.306	0.44	0.5
43	546.69	7.61	0.66	15.78	27.15	196.64	5.79	0.835	0.626	0.94	0.9
44	1653.31	7.61	0.66	34.22	97.85	196.64	5.79	0.651	0.488	0.69	0.7
45	1100.00	7.00	0.43	25.00	62.50	502.50	0.02	0.751	0.563	0.81	0.8
46	1100.00	7.00	0.80	25.00	62.50	502.50	15.01	0.787	0.590	0.84	0.9
47	1653.31	6.39	0.19	15.78	27.15	808.36	24.23	0.589	0.441	0.63	0.7
48	1100.00	6.00	0.43	25.00	62.50	502.50	15.01	0.147	0.671	0.95	0.8
49	1653.31	7.61	0.19	34.22	27.15	196.64	5.79	0.771	0.578	0.81	0.9
50	1100.00	7.00	0.43	25.00	62.50	502.50	15.01	0.787	0.590	0.84	0.9

Table S8. Analysis of variabce table of target NEOs in photolysis degradation experiment

Source	CLO		ACE		IMI		THA	
	F-Value	P-value	F-Value	P-value	F-Value	P-value	F-Value	P-value
Model	13.54	< 0.0001	12.17	< 0.0001	12.25	< 0.0001	18.24	< 0.0001
A-C0	1.24	0.3044	3.15	0.0859	0.23	0.6369	0.26	0.6145
B- pH	0.71	0.3894	21.95	< 0.0001	17.97	0.0003	35.70	< 0.0001
C-Salinity	0.059	0.7558	0.54	0.4664	1.30	0.2652	0.85	0.3646
D- temperature	11.94	0.0013	31.70	< 0.0001	43.48	< 0.0001	46.70	< 0.0001
E-time	27.44	< 0.0001	41.80	< 0.0001	46.14	< 0.0001	67.21	< 0.0001
F- solar radiation	31.25	< 0.0001	45.68	< 0.0001	67.74	< 0.0001	80.22	< 0.0001
G-Humic acids	1.05	0.3110	0.33	0.5690	0.47	0.5002	0.25	0.6196
R^2	0.7593		0.8852		0.93		0.92	
Adep Precision	13.868		14.526		13.644		16.91	

Table S9. The results of target NEOs in the biodegradation degradation experiment

Run	Factor 1	Factor 2	Factor 3	Factor 4	Factor 5	Factor 6	C/C0			
	C0 (ppb)	Time (h)	Temperature (°C)	Ph	Salinity (%)	Humic acids (mg/L)	CLO	THA	IMI	ACE
1	1000.00	63.00	20.00	7.25	0.36	15.01	0.38	0.19	0.42	0.36
2	188.72	99.42	30.22	7.73	0.58	24.59	0.15	0.49	0.38	0.18
3	505.00	63.00	20.00	7.25	0.36	30.00	0.34	0.08	0.48	0.19
4	505.00	6.00	20.00	7.25	0.36	15.01	0.80	0.90	0.82	0.77
5	505.00	63.00	36.00	7.25	0.36	15.01	0.24	0.26	0.27	0.16
6	505.00	63.00	20.00	7.25	0.36	15.01	0.25	0.22	0.66	0.18
7	505.00	63.00	20.00	7.25	0.03	15.01	0.19	0.06	0.71	0.17
8	188.72	26.58	9.78	6.77	0.58	5.43	0.69	0.85	0.75	0.55
9	505.00	63.00	20.00	7.25	0.36	15.01	0.25	0.22	0.66	0.18
10	188.72	26.58	30.22	7.73	0.58	5.43	0.38	0.59	0.47	0.28
11	821.28	99.42	30.22	6.77	0.15	24.59	0.29	0.48	0.59	0.58
12	821.28	99.42	30.22	6.77	0.58	5.43	0.25	0.51	0.61	0.54
13	188.72	26.58	30.22	7.73	0.15	24.59	0.48	0.54	0.40	0.35
14	821.28	26.58	9.78	7.73	0.15	24.59	0.83	0.71	0.55	0.72
15	821.28	26.58	30.22	7.73	0.58	24.59	0.54	0.49	0.28	0.65
16	188.72	99.42	9.78	7.73	0.58	5.43	0.25	0.64	0.55	0.20
17	188.72	99.42	9.78	6.77	0.58	24.59	0.57	0.64	0.59	0.35
18	505.00	120.00	20.00	7.25	0.36	15.01	0.11	0.10	0.31	0.14
19	821.28	99.42	30.22	7.73	0.15	5.43	0.21	0.32	0.41	0.43
20	821.28	99.42	9.78	7.73	0.58	24.59	0.41	0.39	0.66	0.81
21	505.00	63.00	20.00	6.50	0.36	15.01	0.65	0.26	0.71	0.35
22	505.00	63.00	20.00	7.25	0.36	15.01	0.25	0.22	0.66	0.18
23	505.00	63.00	20.00	7.25	0.70	15.01	0.51	0.07	0.47	0.19
24	505.00	63.00	20.00	8.00	0.36	15.01	0.13	0.32	0.40	0.14
25	188.72	26.58	30.22	6.77	0.58	24.59	0.74	0.61	0.56	0.32
26	505.00	63.00	20.00	7.25	0.36	15.01	0.25	0.22	0.66	0.18
27	188.72	99.42	9.78	7.73	0.15	24.59	0.21	0.54	0.52	0.25
28	821.28	26.58	9.78	7.73	0.58	5.43	0.21	0.68	0.72	0.93
29	505.00	63.00	20.00	7.25	0.36	0.02	0.30	0.09	0.68	0.16
30	505.00	63.00	20.00	7.25	0.36	15.01	0.25	0.22	0.66	0.18
31	188.72	99.42	30.22	6.77	0.15	5.43	0.30	0.66	0.55	0.25

32	188.72	26.58	9.78	6.77	0.15	24.59	0.92	0.76	0.64	0.52
33	188.72	26.58	9.78	7.73	0.15	5.43	0.27	0.64	0.49	0.63
34	821.28	99.42	30.22	6.77	0.15	5.43	0.75	0.52	0.63	0.56
35	821.28	99.42	9.78	6.77	0.15	5.43	0.51	0.66	0.74	0.61
36	821.28	26.58	9.78	6.77	0.58	24.59	0.43	0.80	0.78	0.83
37	821.28	26.58	30.22	6.77	0.15	5.43	0.62	0.40	0.76	0.70
38	505.00	63.00	4.00	7.25	0.36	15.01	0.80	0.77	0.91	0.24
39	10.00	63.00	20.00	7.25	0.36	15.01	0.36	0.58	0.21	0.45
40	505.00	63.00	20.00	7.25	0.36	15.01	0.25	0.22	0.66	0.18

Table S10. Analysis of variance results of target NEOs in biodegradation experiment

Source	CLO		ACE		IMI		THA	
	F-Value	P-value	F-Value	P-value	F-Value	P-value	F-Value	P-value
Model	6.00	<0.0001	6.92	<0.0001	9.70	<0.0001	8.72	<0.0001
A-C0	1.05	0.3146	3.76	0.0610	31.60	<0.0001	9.68	0.0053
B- Time	40.51	<0.0001	3.43	0.0730	27.46	<0.0001	15.32	0.0008
C-Temperature	16.35	0.0005	18.48	0.0001	8.56	0.0078	21.32	0.0001
D- pH	8.03	0.0092	14.84	0.0005	0.95	0.3401	1.48	0.2380
E-Salinity	1.48	0.2348	0.16	0.6915	0.12	0.7305	0.90	0.3537
F- Humic acids	7.86	0.0098	2.98	0.0938	0.06	0.8081	0.11	0.7425
R^2	0.7894		0.5572		0.8823		0.8819	
Adep Precision	10.338		11.384		10.142		10.297	

$$C_{CLO} = 0.71 + 0.067 * \mathrm{A} - 0.094 * \mathrm{B} - 0.095 * \mathrm{C} + 1.63\mathrm{E} - 3 * \mathrm{D} + 1.85\mathrm{E} - 3 * \mathrm{E} - 7.17\mathrm{E} - 3 * \mathrm{F} + 0.086 * \mathrm{A} * \mathrm{B} - 0.030 * \mathrm{A} * \mathrm{D} - 0.02 * \mathrm{A} * \mathrm{E} + 0.077 * \mathrm{B} * \mathrm{C} + 0.035 * \mathrm{C} * \mathrm{D} - 0.012 * \mathrm{C} * \mathrm{F} + 0.019 * \mathrm{D} * \mathrm{E} + 0.013 * \mathrm{E} * \mathrm{F} + 0.034 * C^2 - 0.028 * D^2 - 0.022 * E^2 - 0.097 * F^2 \quad \text{(S1)}$$

$$C_{THA} = 0.46 - 0.038 * \mathrm{A} - 0.027 * \mathrm{B} - 0.028 * \mathrm{C} - 0.025 * \mathrm{D} + 1.03\mathrm{E} - 3 * \mathrm{E} - 7.26\mathrm{E} - 3 * \mathrm{F} - 0.01 * \mathrm{A} * \mathrm{C} + 0.013 * \mathrm{A} * \mathrm{D} + 0.014 * \mathrm{A} * \mathrm{F} - 0.034 * \mathrm{B} * \mathrm{D} + 0.021 * \mathrm{B} * \mathrm{E} + 0.032 * \mathrm{C} * \mathrm{D} - 0.064 * \mathrm{C} * \mathrm{E} + 0.018 * \mathrm{C} * \mathrm{F} - 0.015 * \mathrm{D} * \mathrm{E} + 0.035 * \mathrm{D} * \mathrm{F} - 0.11 * A^2 - 0.019 * B^2 - 0.025 * C^2 - 0.030 * D^2 - 0.031 * E^2 - 0.030 * F^2 \quad \text{(S2)}$$

$$C_{ACE} = 0.35 - 0.13 * \mathrm{A} - 0.03 * \mathrm{B} - 0.14 * \mathrm{C} - 0.042 * \mathrm{D} - 0.031 * \mathrm{E} + 0.026 * \mathrm{F} - 0.056 * \mathrm{A} * \mathrm{B} - 0.031 * \mathrm{A} * \mathrm{D} + 0.084 * \mathrm{A} * \mathrm{E} - 0.11 * \mathrm{A} * \mathrm{F} - 0.077 * \mathrm{B} * \mathrm{D} + 0.022 * \mathrm{B} * \mathrm{E} - 0.056 * \mathrm{B} * \mathrm{F} - 0.068 * \mathrm{C} * \mathrm{E} + 0.027 * \mathrm{D} * \mathrm{E} + 0.096 * \mathrm{D} * \mathrm{F} + 0.03 * \mathrm{E} * \mathrm{F} \quad \text{(S3)}$$

$$C_{IMI} = 0.47 + 0.03 * \mathrm{A} - 0.05 * \mathrm{B} - 0.12 * \mathrm{C} - 0.064 * \mathrm{D} - 0.057 * \mathrm{E} - 0.016 * \mathrm{F} - 0.058 * \mathrm{A} * \mathrm{B} - 0.069 * \mathrm{A} * \mathrm{D} + 0.046 * \mathrm{A} * \mathrm{E} - 0.12 * \mathrm{B} * \mathrm{D} + 0.13 * \mathrm{B} * \mathrm{E} - 0.1 * \mathrm{B} * \mathrm{F} + 0.093 * \mathrm{C} * \mathrm{D} - 0.10 * \mathrm{B} * \mathrm{F} + 0.093 * \mathrm{C} * \mathrm{D} - 0.10 * \mathrm{C} * \mathrm{E} + 0.045\mathrm{C} * \mathrm{F} - 0.032 * \mathrm{D} * \mathrm{F} + 0..65 * \mathrm{E} * \mathrm{F} \quad \text{(S4)}$$

where C_{CLO}, C_{THA}, C_{ACE}, and C_{IMI} were the concentrations of CLO, THA, ACE, and IMI at time t, respectively; A, B, C, D, E, and F indicated that the initial concentrations of NEOs (ppb), the reaction time (h), temperature (°C), pH, Salinity, and humic acids (mg/L), respectively.

$$C_{CLO} = 0.77 - 0.016 * \mathrm{A} - 0.014 * \mathrm{B} - 4.902\mathrm{E} - 3 * \mathrm{C} - 0.055 * \mathrm{D} - 0.085 * \mathrm{E} - 0.087 * \mathrm{F} - 0.016 * \mathrm{G} + 0.038 * \mathrm{A} * \mathrm{C} - 0.045 * \mathrm{A} * \mathrm{F} - 0.14 * B^2 \quad \text{(S5)}$$

$$C_{THA} = 0.60 - 3.346\mathrm{E} - 3 * \mathrm{A} - 0.041 * \mathrm{B} - 4.243\mathrm{E} - 3 * \mathrm{C} - 0.048 * \mathrm{D} - 0.060 * \mathrm{E} - 0.063 * \mathrm{F} - 4.52\mathrm{E} - 3 * \mathrm{G} + 0.028 * \mathrm{A} * \mathrm{C} - 0.028 * \mathrm{A} * \mathrm{F} - 0.02 * \mathrm{B} * \mathrm{F} + 0.018 * \mathrm{E} * \mathrm{F} - 0.029 * B^2 - 0.035 * D^2 - 0.032 * F^2 - 0.030 * G^2 \quad \text{(S6)}$$

$$C_{ACE} = 0.84 - 0.021 * \mathrm{A} - 0.056 * \mathrm{B} - 8.71\mathrm{E} - 3 * \mathrm{C} - 0.069 * \mathrm{D} - 0.079 * \mathrm{E} - 0.081 * \mathrm{F} - 6.96\mathrm{E} - 3 * \mathrm{G} + 0.018 * \mathrm{A} * \mathrm{B} + 0.036 * \mathrm{A} * \mathrm{C} + 0.021 * \mathrm{A} * \mathrm{D} - 0.035 * \mathrm{A} * \mathrm{F} - 0.025 * \mathrm{B} * \mathrm{F} - 0.013 * \mathrm{C} * \mathrm{E} + 0.014 * \mathrm{D} * \mathrm{F} + 0.02 * \mathrm{E} * \mathrm{F} - 0.072 * A^2 - 0.033 * D^2 - 0.029 * F^2 \quad \text{(S7)}$$

$$C_{IMI} = 0.90 - 5.51\mathrm{E} - 3 * \mathrm{A} - 0.048 * \mathrm{B} - 0.013 * \mathrm{C} - 0.075 * \mathrm{D} - 0.076 * \mathrm{E} - 0.091 * \mathrm{F} - 7.69\mathrm{E} - 3 * \mathrm{G} + 0.025 * \mathrm{A} * \mathrm{B} + 0.038 * \mathrm{A} * \mathrm{C} + 0.022 * \mathrm{A} * \mathrm{D} - 0.041 * \mathrm{A} * \mathrm{F} - 0.025 * \mathrm{B} * \mathrm{F} - 0.012 * \mathrm{C} * \mathrm{E} + 0.011 * \mathrm{D} * \mathrm{F} + 0.019 * \mathrm{E} * \mathrm{F} - 4.53\mathrm{E} - 3 * \mathrm{F} * \mathrm{G} - 0.016 * A^2 - 0.056 * B^2 - 0.013 * C^2 - 0.031 * D^2 - 0.037 * E^2 - 0.029 * F^2 - 0.024 * G^2 \quad \text{(S8)}$$

where C_{CLO}, C_{THA}, C_{ACE}, and C_{IMI} were the concentrations of CLO, THA, ACE, and IMI at time t, respectively; A, B, C, D, E, F, and G indicated that the initial concentrations of NEOs (ppb), pH, Salinity, temperature (°C), the solar time (min), solar radiation (W), and humic acids (mg/L), respectively.

$$C_{CLO} = 0.38 - 0.028 * \mathrm{A} - 0.17 * \mathrm{B} - 0.11 * \mathrm{C} - 0.077 * \mathrm{D} + 0.034 * \mathrm{E} + 0.075 * \mathrm{F} + 0.065 * \mathrm{A} * \mathrm{B} + 0.055 * \mathrm{A} * \mathrm{C} - 0.11 * \mathrm{A} * \mathrm{E} - 0.032 * \mathrm{A} * \mathrm{F} - 0.042 * \mathrm{B} * \mathrm{D} - 0.087 * \mathrm{B} * \mathrm{F} - 0.077 * \mathrm{C} * \mathrm{F} + 0.073 * \mathrm{D} * \mathrm{E} + 0.088 * \mathrm{D} * \mathrm{F} \quad \text{(S9)}$$

$$C_{THA} = 0.18 - 0.072 * \mathrm{A} - 0.089 * \mathrm{B} - 0.11 * \mathrm{C} - 0.028 * \mathrm{D} + 0.022 * \mathrm{E} + 7.85\mathrm{E} - 3 * \mathrm{F} - 0.027 * \mathrm{A} * \mathrm{D} - 0.026 * \mathrm{A} * \mathrm{E} + 0.046 * \mathrm{B} * \mathrm{C} - 0.033 * \mathrm{B} * \mathrm{D} - 0.035 * \mathrm{B} * \mathrm{E} - 0.047 * \mathrm{B} * \mathrm{F} + 0.024 * \mathrm{D} * \mathrm{E} + 0.090 * A^2 + 0.14 * B^2 + 0.14 * C^2 + 0.052 * D^2 - 0.040 * E^2 \quad \text{(S10)}$$

$$C_{ACE} = 0.57 + 0.044 * \mathrm{A} - 0.042 * \mathrm{B} - 0.097 * \mathrm{C} - 0.087 * \mathrm{D} - 9.04\mathrm{E} - 3 * \mathrm{E} - 0.039 * \mathrm{F} \quad \text{(S11)}$$

$$C_{IMI} = 0.17 + 0.12 * \mathrm{A} - 0.11 * \mathrm{B} - 0.063 * \mathrm{C} - 0.022 * \mathrm{D} + 7.53\mathrm{E} - 3 * \mathrm{E} + 5.38\mathrm{E} - 3 * \mathrm{F} + 0.029 * \mathrm{A} * \mathrm{B} + 0.036 * \mathrm{A} * \mathrm{E} + 0.025 * \mathrm{B} * \mathrm{C} + 0.032 * \mathrm{B} * \mathrm{D} - 0.029 * \mathrm{C} * \mathrm{D} - 0.028 * \mathrm{C} * \mathrm{E} + 0.036 * \mathrm{E} * \mathrm{F} + 0.11 * A^2 + 0.13 * B^2 + 0.031 * C^2 + 0.0548 * D^2 \quad \text{(S12)}$$

where C_{CLO}, C_{THA}, C_{ACE}, and C_{IMI} were the concentrations of CLO, THA, ACE, and IMI at time t, respectively; A, B, C, D, E, and F indicated that the initial concentrations of NEOs (ppb), the reaction time (h), temperature (°C), pH, Salinity, and humic acids (mg/L), respectively.

Figure S1

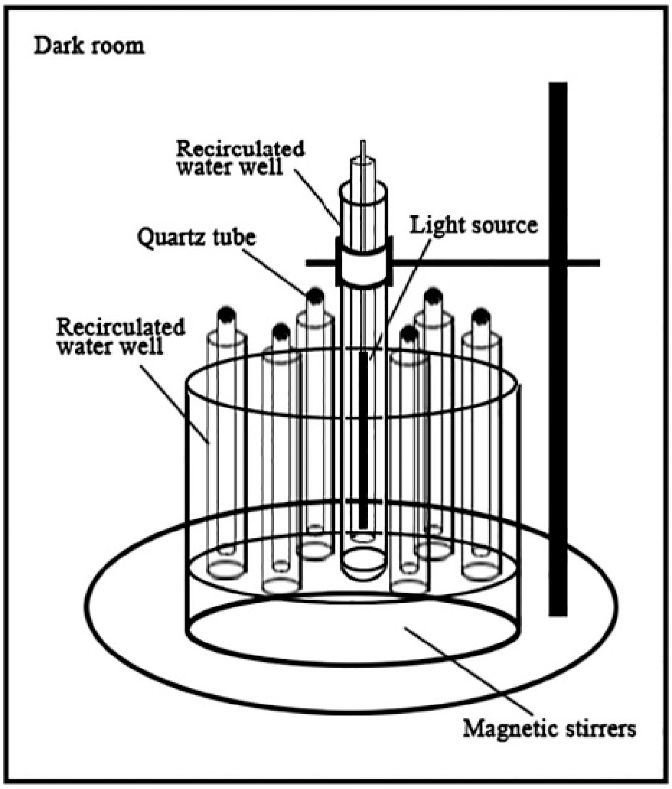

Figure S1. Rotary photochemical reactor
